# First report on the application of near-infrared spectroscopy to predict the age of *Aedes albopictus* Skuse

**DOI:** 10.1038/s41598-018-27998-7

**Published:** 2018-06-25

**Authors:** Maggy T. Sikulu-Lord, Gregor J. Devine, Leon E. Hugo, Floyd E. Dowell

**Affiliations:** 1 Mosquito Control Laboratory, QIMR Berghofer Medical Research Institute, 300 Herston Road, Herston, Queensland 4006 Australia; 2 Queensland Alliance for Agriculture and Food Innovation, The University of Queensland, 306 Carmody Road, St Lucia, Queensland 4072 Australia; 3 USDA, Agricultural Research Service, Center for Grain and Animal Health Research, 1515 College Avenue, Manhattan, KS 66502 USA

## Abstract

To date, no methodology has been described for predicting the age of *Aedes albopictus* Skuse mosquitoes, commonly known as Asian tiger mosquitoes. In this study, we report the potential of near-infrared spectroscopy (NIRS) technique for characterizing the age of female laboratory reared *Ae*. *albopictus*. Using leave-one-out cross-validation analysis on a training set, laboratory reared mosquitoes preserved in RNA*later* for up to a month were assessed at 1, 3, 7, 9, 13, 16, 20 and 25 days post emergence. Mosquitoes (N = 322) were differentiated into two age classes (< or ≥ 7 days) with 93% accuracy, into three age classes (<7, 7–13 and >13 days old) with 76% accuracy, and on a continuous age scale to within ±3 days of their actual average age. Similarly, models predicted mosquitoes (N = 146) excluded from the training model with 94% and 71% accuracy to the two and the three age groups, respectively. We show for the first time that NIRS, with an improved spectrometer and fibre configuration, can be used to predict the age of laboratory reared female *Ae*. *albopictus*. Characterization of the age of *Ae*. *albopictus* populations is crucial for determining the efficacy of vector control interventions that target their survival.

## Introduction

Over the last three decades, the mosquito *Aedes albopictus* Skuse commonly referred to as the Asian tiger mosquito, has rapidly spread to new regions; notably Europe, the Americas, Africa and the Caribbean^1^. *Ae*. *albopictus* is a competent vector of dengue, Zika and chikungunya viruses^2–4^. With an estimated 390 million cases each year in over 100 countries, dengue has increasingly become a public health problem over the last five decades putting half of the world’s population at risk^5–7^. *Ae*. *albopictus* is also a highly competent vector of chikungunya virus which has been responsible for major outbreaks on Reunion Island^8^, Gabon^9^, the Caribbean, South and Central America^10^ and more recent outbreaks in Italy^11–13^. The ability of *Ae*. *albopictus* to thrive in newly invaded regions is largely due to a broad thermal tolerance. Unlike the primary dengue vector *Ae*. *aegypti*, *Ae*. *albopictus* is not limited to tropical regions, and its invasion has already redefined the distribution of arboviral diseases, for example, bringing chikungunya to temperate regions of Europe^12,13^. There is a widespread concern about what the increased distribution of the species means for the burden of several mosquito-borne arboviruses^3^.

Mosquito survival is a critical determinant of its vectorial capacity. Only those mosquitoes that have survived longer than the extrinsic incubation period of the pathogen they are carrying (defined as the interval between the acquisition of an infectious agent by a vector and the vector’s ability to transmit the agent to a new, susceptible host^14^) can transmit diseases. According to the vectorial capacity model^15,16^, a slight change in mosquito survival can lead to exponentially larger changes in pathogen transmission. Because mosquito age plays a significant role in disease transmission, it is crucial to establish the survival characteristics of *Ae*. *albopictus* particularly in newly invaded areas.

To date, no technique has been described to predict the age and or survivorship of *Ae*. *albopictus*. While classical age grading techniques such as those described by Detinova^17^ and Polovodova^18^ to determine the gonotrophic history of females mosquitoes could theoretically be applied to *Ae*. *albopictus*, these techniques are laborious, technically demanding and can be highly inaccurate^19^. Moreover it remains unclear whether these classical age grading techniques are applicable to *Ae*. *albopictus*.

Near-infrared spectroscopy (NIRS) has been used to predict the age of the major African malaria vectors *Anopheles gambiae* and *An*. *arabiensis*^20–25^, and to predict the age of wild type *Ae*. *aegypti* and *Ae*. *aegypti* infected with *Wolbachia*^26,27^. NIRS has also been used to detect *Wolbachia*^28^ and Zika virus^29^ infections in *Ae*. *aegypti*. This technique detects molecular vibrations resulting from brief exposure of a sample to light in the near infrared spectrum (approximately 700–2500 nm). The spectrum is obtained from a <10 s scan from the head and thorax, providing a characteristic spectral signature. The spectral signature is defined by the composition and concentration of chemical compounds within the samples being analysed. The NIRS age grading technique is rapid, non-destructive, and cost effective.

NIRS can classify mosquitoes into groups of young (<7 days old) and old (≥7 days old) mosquitoes^23,25^, representing those less likely to be infectious and those potentially infectious, respectively, with accuracies exceeding 90%. Here we describe for the first time, the use of NIRS for determining the age of laboratory reared female *Ae*. *albopictus*.

## Results

We tested the capability of NIRS to predict the age of *Ae*. *albopictus* preserved in RNA*later* for a month. We analysed spectra collected from the heads and thoraces of eight age groups of female laboratory reared mosquitoes using a colony established with collections from the Torres Strait, northern Australia. To develop age prediction models, we used leave-one-out cross-validation analysis of a training set, where one sample is removed from the population and the rest of the samples are used to predict the age of the removed sample and the process is repeated for all the samples. The model was validated on a subset of mosquito samples that were excluded from the training model. NIRS allowed a rapid age identification of these mosquitoes into multiple age categories that can be used to define their potential for transmitting pathogenic arboviruses.

**Figure 1:**
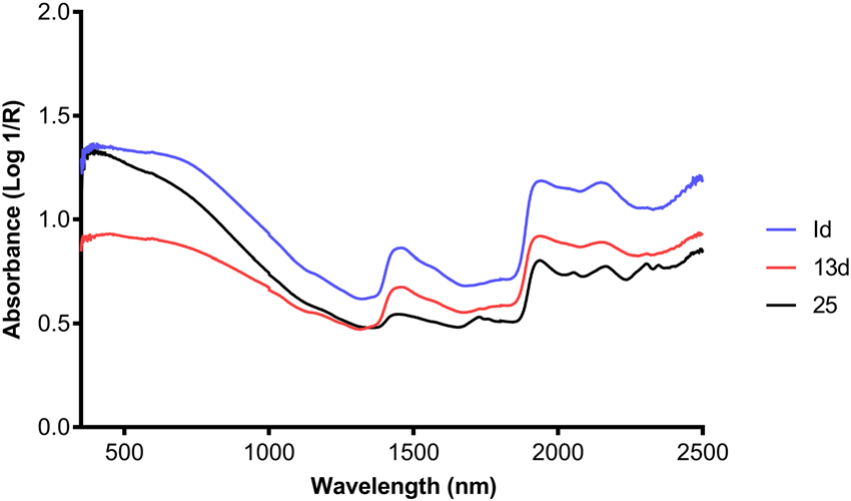
Typical average spectra of 1 d, 13 d and 25 d old female *Ae*. *albopictus* collected from their heads and thoraces.

**Figure 2:**
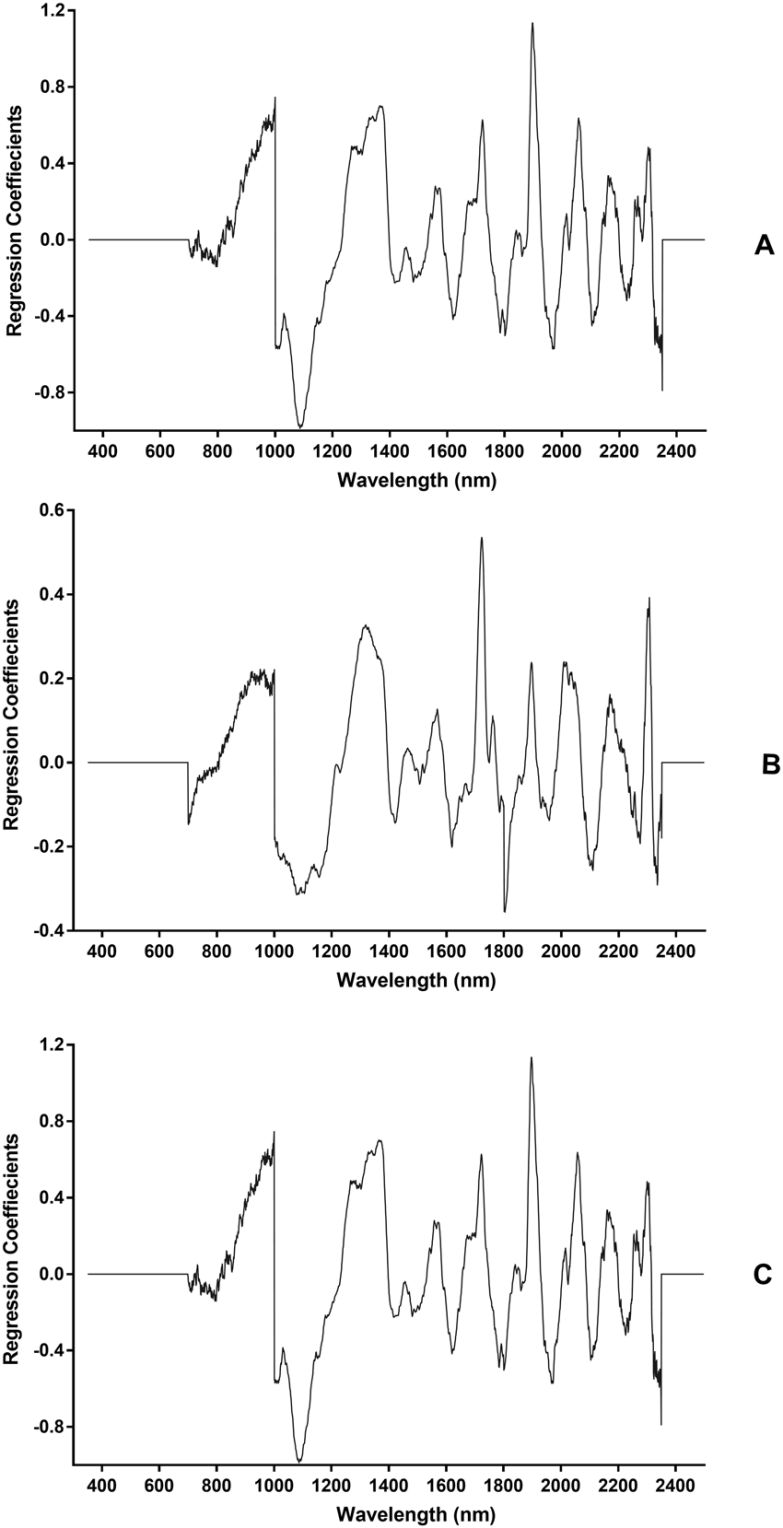
Regression coefficient plots with 10 factors for age prediction of female *Ae. albopictus* mosquitoes on a continuous age scale (Panel A), prediction into two age groups (Panel B) and prediction into three age groups (Panel C).

Typical spectra collected from the head and thorax of *Ae*. *albopictus* are shown in Fig. 1 and regression coefficient plots used for predicting the age of female *Ae*. *albopictus* into various age groups are shown in Fig. 2. The spectrometer and fibre-optic probe used in this study have improvements in the light source, optics, electronics, and fibre material resulting to an improved spectra with less noise compared to those reported in previous studies^21,23–26,28,30^.

**Figure 3:**
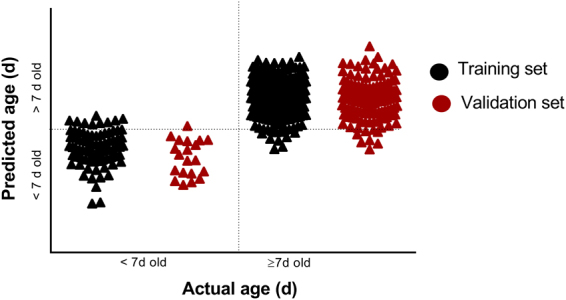
The accuracy of NIRS for predicting female *Ae*. *albopictus* as < or ≥7 d days old age groups for training and validation data sets. The horizontal dotted line indicates the cut off point for correct prediction whereas the vertical dotted line separates <7 days from ≥7 days old mosquitoes.

**Figure 4:**
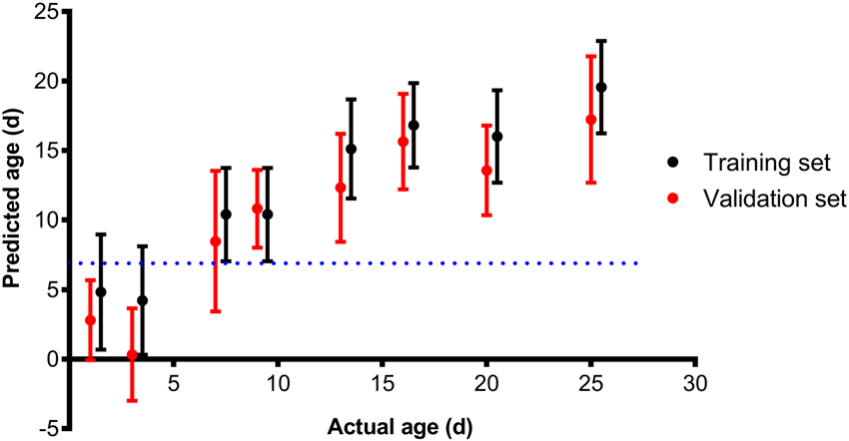
Mean age prediction of female *Ae*. *albopictus* using the training and validation sets. Error bars indicate standard deviation and the blue dotted lines indicates the 7 day cut off point that differentiates <7 from ≥7 days old mosquitoes.

The total number of mosquitoes used in the training set was 322 and the model was tested on a total of 146 mosquitoes. Using cross-validation analysis, female *Ae*. *albopictus* were predicted into < or ≥7 days age groups with 93% accuracy (Table 1 and Fig. 3), into <7, 7–13 and >13 days age groups with 76% accuracy (Table 1) or generally into ±3 days of the actual average age (Table 2 and Fig. 4). Similarly, mosquitoes that were excluded from the model were predicted into two and three age groups with 94% and 71% accuracy, respectively, (Table 1) and to within ±3 days of their actual average age (Table 2 and Fig. 4). The predicted age of very young mosquitoes (one and three day old) was significantly different from all other age groups (Table 2). Results from analysis of variance (ANOVA) indicated mosquitoes could generally be clustered into three statistically distinct age groups (<7, 7–13 and >13 d old), as indicated in Table 2.

**Table 1:** Percent predictive accuracy of NIRS for determining the age of female *Ae*. *albopictus* into specific age groups.

% Predictive accuracy per age group										
Sample set	**Age group** **(Days)**	1	3	7	9	13	16	20	25	**Overall % accuracy**
Training set	<7, ≥7	87.5	85	95	90	97.5	97.5	97.5	100	93.5
	<7, 7–13, >13	70	72.5	85	90	72.5	75	50	85	76
Validation set	<7, ≥7	89	100	83	80	95	100	93	100	94.5
	>7, 7–13 > 13	78	90	67	80	77	50	28	64	70.5

**Table 2:** The mean age of *Ae*. *albopictus* in the training and validation sets as predicted by NIRS. Mean predicted age followed by the same letter are not statistically significant at P < 0.05 when using Tukey post hoc test Actual and mean predicted ages shown are in days.

Actual age	Training set (N = 322)		Validation set (N = 146)	
	Mean predicted age [95% CI]	SD	Mean predicted age [95% CI]	SD
1	4.3^a^ [3.0–5.6]	4.1	2.8^a^ [0.6–5.0]	2.8
3	3.7^a^ [2.4–4.9]	3.9	0.3^a^ [−1.8–2.5]	3.3
7	9.9^b^ [8.8–10.9]	3.3	8.5^b^ [5.2–11.6]	5.0
9	10.6^b^ [9.1–12.0]	4.4	10.8^b,c^ [7.3–14]	2.7
13	14.6^c^ [13.4–15.7]	3.5	12.3^c^ [11.3–13.3]	3.8
16	16.3^c^ [15.3–17.2]	3.0	15.6^c^ [12.7–18.5]	3.4
20	15.5^c^ [14.4–16.5]	3.3	13.7^c^ [11.7–15.4]	3.2
25	19.1^d^ [18.0–20.1]	3.3	17.2^c^ [15.3–19.1]	4.5

The highest classification accuracy of 94% was achieved when mosquitoes were simply grouped into two age groups (<or ≥7 days age group). Seven days was chosen because it provided the highest classification accuracy of mosquitoes into two age groups and because dissemination efficiency of some viruses such as chikungunya in *Ae*. *albopictus* has been reported to peak at 7 days post infection^31^. Mosquitoes were less accurately predicted if three or more age groups were used in the analysis. Additionally, the younger age groups (1–3 d and 7–9 d old mosquitoes) were more easily differentiated from the older age groups (>13 days old mosquitoes) than they were from each other. This could be due to minimal biochemical changes occurring between closely related age groups as previously described^32,33^.

The age prediction accuracy of female *Ae*. *albopictus* was slightly better than the prediction for laboratory reared female *Ae*. *aegypti*^26^ or female *An*. *gambiae* mosquitoes reared under laboratory^23,25^ or semi-field^30^ environments. This could be attributed to innate differences in biochemical composition or improved spectra collected using the current spectrometer and fibre configuration compared to those used in previous studies. Peaks around 1100, 1300, 1550, 1600, 1700, 1900, 1950, 2050, 2150 and 2300 nm appeared to be the major spectral regions distinguishing the ages of *Ae*. *albopictus* (Fig. 2). These regions comprise C-H and N-H functional groups and have previously been shown to play a role in age grading *Aedes* and Anopheline mosquitoes^32,34–37^.

## Discussion

Characterising the age structure of *Ae*. *albopictus* is a crucial determinant of the efficacy of mosquito control interventions, particularly those designed to reduce mosquito survival. Defining mosquito population age structure is also critical as it helps to define the risk of arbovirus transmission^38^. This is due to the fact that mosquitoes must live long enough to allow for the pathogens they acquire through a blood meal to replicate in mesenteronal epithelial cells, disseminate to other tissues and finally to the salivary glands where they can be transmitted to a susceptible host during blood feeding^14^. *Ae*. *albopictus* is a vector of several important human pathogens including dengue, Zika, yellow fever and chikungunya viruses. Vector control tools are designed to interrupt the normal development of these viruses inside the mosquito by reducing the survival of the mosquito vector. For example, pathogenic bacteria (*Wolbachia*) shortens mosquito’s lifespan, reducing the time available for the virus to develop within the mosquito. Alternatively, *Wolbachia* induces resistance against dengue and chikungunya infection therefore blocking their transmission^39,40^.

To date, age grading options for *Ae*. *albopictus* remain poorly investigated. The classical age grading techniques based on gonotrophic history of mosquitoes^17,18^ have not been assessed for *Ae*. *albopictus*. Similarly, the potential of more accurate age grading techniques developed for *Ae*. *aegypti* such as analysis of changes in abundance of transcriptional profiles^41,42^ and cuticular hydrocarbons^43^ have not been attempted for age grading *Ae*. *albopictus*. Changes in abundance of protein age biomarkers as a potential age grading technique for *Ae*. *albopictus* has only been recently described^44^ and it’s still early in development.

Here we describe an easy to use and rapid method for age grading *Ae*. *albopictus*. NIRS is field-portable, cost effective and could be useful in comparative studies to establish longevity of *Ae*. *albopictus* in various regions as well as its survival across different seasons following invasion. The technique allows hundreds of samples to be analysed in a day without consuming any reagents. After training models have been established, only minimal computer skills are required.

Although the cost for buying NIRS instruments are high ($60,000), the cost can easily be justified through the programmatic use of the technique for multiple applications including species identification, age grading, and infection detection. In comparison with the polymerase chain reaction technique which costs ~10 dollars/mosquito, the costs of buying the NIRS instrument could easily be recovered after analysis of approximately 10,000 samples.

This study provides a platform for the future development of this technique for investigating the age of *Ae*. *albopictus*. Further work is required to establish the technique’s accuracy under varying environmental conditions to account for factors such as varying diet, temperature and humidity. Following assessments on laboratory mosquitoes, the next phase of validation for age grading techniques can involve comparison of predictions of wild caught specimens against predictions made with a standard age grading method. However, as there are no alternative age grading techniques for *Ae*. *albopictus*, we recommend validating NIRS using wild *Ae*. *albopictus* larvae/pupae reared to known ages in field cages.

We conclude that the use of NIRS for age prediction of *Ae*. *albopictus* is consistent with accuracies previously reported for other mosquito species including *Ae*. *aegypti* and *An. gambiae*, thereby extending the range of mosquito vectors that can be age graded using this technology.

## Materials and Methods

### Ethics statement

Ethics approval for routine blood feeding of mosquito colonies was obtained from QIMR Berghofer Medical Research Institute (QIMR HREC P1162). All experiments were carried out in accordance with relevant human ethics guidelines and regulations. Informed consent was obtained from volunteers involved in blood feeding and blood feeding volunteers were free to withdraw their participation at any time.

### Mosquito rearing

Mosquitoes were obtained from a colony of *Ae*. *albopictus* established in the insectary at QIMR Berghofer Medical Research Institute from materials collected on Hammond Island, Torres Strait, Australia in 2014. These mosquitoes are routinely maintained at 27 °C, 70% humidity with 12:12 hr day:night lighting and 30 min dawn/dusk periods. Approximately 50 larvae were reared in aged tap water in plastic containers (17 × 12 × 7 cm). Larvae were provided ground Tetramin tropical flakes fish food at the following rate; first and second instar larvae were fed on 0.16 mg/larva/day whereas 3rd and 4^th^ instar larvae were fed on 0.32 mg/larva/day. Approximately 200 pupae were transferred into small cages measuring 31 × 20 × 20 cm for adult emergence. Adult mosquitoes were given a 48 hr time window to emerge into the cage before the remaining pupae were removed. Thereafter, adult female mosquitoes were collected 24 hr after emergence and at 3, 7, 9, 13, 16, 20 and 25 days post emergence. Mosquitoes were supplied with 10% sucrose *ad libitum*, and blood fed on a human volunteer for 15 min every 7 d. Female *Ae*. *albopictus* were knocked down with carbon dioxide and stored whole in RNA*later®* solution for about 4 weeks before scanning^21^.

### Mosquito scanning using NIR spectrometer

Residual RNA*later* was removed from the mosquito specimens by gently blotting the mosquito with a paper towel prior to scanning. At least 48 mosquitoes at each age were scanned as described previously^23^ using a LabSpec 4S*i* NIR spectrometer (ASD Inc, Boulder, CO) and a 3.2 mm-diameter bifurcated fibre-optic probe which contained a single 600 micron collection fibre surrounded by six 600 micron illumination fibres.

### Data Analysis

All spectra were analysed using partial least squares (PLS). Analysis was limited to within the near-infrared region between 700–2350 nm to exclude a region of high spectral ‘noise’ observed from 2350 to 2500 nm. At least 40 mosquitoes were included in the analysis at each age. Mosquitoes were divided into a training set (N = 322) and a validation set (N = 146). The training set classification accuracy along with the predicted residual error sum of squares (PRESS) and regression coefficient plots were used to select the number of factors used in the training set for predicting the ages of mosquitoes in the validation set.

Three classification models, each with 10 factors chosen from the cross-validation analysis, were developed to predict mosquitoes into 1) two groups (< or ≥7 days old), 2) three groups (< 7, 7–13 and >13 days old) and 3) on a continuous age scale. Ten factors were chosen as they provided maximum accuracy with minimal noise in the regression coefficient graph when compared to models with >10 factors. Each of the models was then applied to predict the age of samples in the validation set. We tested whether the mean predicted ages differed significantly between age groups using ANOVA and Tukey post hoc analysis in Statistical Package for Social Sciences 22 (IBM, Armonk, NY).

### Data availability

The datasets generated and/or analysed during the current study are available from the corresponding author.
